# Acquiring New Factual Information: Effect of Prior Knowledge

**DOI:** 10.3389/fpsyg.2018.01734

**Published:** 2018-09-24

**Authors:** Haoyu Chen, Xueling Ning, Lingwei Wang, Jiongjiong Yang

**Affiliations:** School of Psychological and Cognitive Sciences, Beijing Key Laboratory of Behavior and Mental Health, Peking University, Beijing, China

**Keywords:** semantic memory, conceptual representation, prior knowledge, episodic memory, memory consolidation

## Abstract

One influential theory on object knowledge is feature-based model, which proposes that the object knowledge is organized by different feature types, such as sensory/perceptual and motor/functional ones. Previous studies have shown that prior knowledge enhances the processes of acquiring and remembering relevant information. However, whether the effect of prior knowledge is applied to different types of conceptual information over time remains unclear. In this study, we addressed this question by testing memory of different types of object features at various retention intervals. The level of prior knowledge was manipulated as object features from familiar and unfamiliar categories. In Experiments 1 and 2, sentences that described the perceptual and functional features of new words were presented. Sentences with episodic features were additionally presented in Experiment 2. The participants were then tested with recognition (Experiment 1) and recall (Experiment 2) tasks at different retention intervals. The results showed that prior knowledge enhanced memory for perceptual features but not for functional and episodic features. Such enhancement depended on the recollection process. In addition, the effect of prior knowledge on perceptual features remained stable over time. This study clarified how different types of new factual information were acquired and maintained and highlighted the importance of prior knowledge in acquiring new conceptual knowledge with the passage of time.

## Introduction

We have a large amount of general knowledge about every-day objects. This type of memory is referred to as semantic memory or conceptual knowledge (Patterson et al., 2007; Binder and Desai, 2011). One influential theory in this field is feature-based model. It proposes that conceptual knowledge is organized by different attributes or features, such as perceptual and motor/functional, and processing these features relies on different brain systems (Warrington and McCarthy, 1983; Warrington and Shallice, 1984; Barsalou et al., 2003; Martin, 2007; Mahon and Caramazza, 2009; Gainotti et al., 2013; Binder et al., 2016). For example, when we see flowers, we obtain their perceptual attributes, such as color, shape, appearance, and odor. Their functional features, such as where they grow and what particular function they serve (e.g., scissors can be used for cutting papers), are also obtained. So the perceptual features are defined as information from different perceptual modalities (e.g., visual, auditory), whereas the functional features are defined as abstract and propositional properties (e.g., where objects are typically found, their social significance, and context use; Warrington and Shallice, 1984; Martin and Chao, 2001; Canessa et al., 2008).

One interesting question we focused on in this study is how the conceptual knowledge of new objects is acquired and maintained over time (Martin, 2007; Binder and Desai, 2011). Previous studies have suggested that prior knowledge availability is important for acquiring new semantic information. Information with prior knowledge is more easily remembered than information without it (Alba and Hasher, 1983). The effect of prior knowledge has been found when new stimuli are congruent with the knowledge of category (Maguire and Frith, 2004; DeWitt et al., 2012; Hennies et al., 2016), academic information (Brandt et al., 2005; van Kesteren et al., 2014; Brod et al., 2016), Star Trek (Long and Prat, 2002), and football (Rawson and Van Overschelde, 2008). For example, in a study of van Kesteren et al. (2014), students in biology and education backgrounds learned new factual sentences that were either related or unrelated to their pre-existing conceptual knowledge. Twenty four hours later, they were tested. The results showed that memory related to their familiar knowledge was significantly higher than chance-level, whereas prior knowledge-unrelated memory was not. In addition, the effect of prior knowledge is driven by recollection process (Long and Prat, 2002; Brandt et al., 2005; Toth et al., 2011). Brandt et al. (2005) enrolled participants in radiography and psychology domains and asked them to learn new words in the two domains. The results showed that memory performance was higher for familiar than for unfamiliar academic words, and this effect was attributed to recollection rather than familiarity process. The findings suggested that prior knowledge provides a semantic context that increases the availability of details supporting later recollection.

However, as the conceptual knowledge is composed of perceptual and functional features, it remains unclear whether the two types of features are acquired and maintained in the same manner, and whether their memory is modulated by prior knowledge. Consider the difference between the perceptual and functional features. Perceptual information represents how an object looks, smells and sounds (Warrington and Shallice, 1984; Martin, 2007), and these features are intrinsic to the entity or object itself. In contrast, functional features are extrinsic because how an object is used and what it is used for are based on relationships between the object and something else, such as actions performed on the object by some agents (Barr and Caplan, 1987; Blumenthal et al., 2017). As the perceptual features are intrinsic to the object, they are more associated with the preexisting conceptual knowledge system. Thus, when compared to the functional features, encoding and retrieving new perceptual features can elicit more spreading activation throughout a preexisting network (Anderson, 1983) and lead to more elaborative process (Rawson and Van Overschelde, 2008; Rawson et al., 2008).

From the neural perspective, the object concepts are represented in the brain as distributed networks in areas involved in processing perceptual or functional knowledge. Different cortical regions are shown to represent sensory-motor/functional features (Martin, 2007), such as color, shape, visual motion, sound, and manipulation (e.g., Fernandino et al., 2016), and socially-related features (Lin et al., 2018). Moreover, it is suggested that the hippocampus is important for learning functional but not perceptual features of conceptual knowledge. Blumenthal et al. (2017) showed that a developmental amnesic patient generated fewer functional features than controls due to hippocampus damage but produced as many perceptual features as controls. On the other hand, studies have suggested that information congruent to prior knowledge can be easily assimilated into the knowledge system without depending heavily on the hippocampus (Tse et al., 2007; van Kesteren et al., 2010). Therefore, perceptual features are less dependent on the hippocampus than functional features, but instead they are closely associated with prior knowledge, so it is possible that memory for them is enhanced when the prior knowledge is available. By contrast, functional features are more dependent on the hippocampus and associated with contexts in which an object is encountered, so it is possible that memory for them is less likely modulated by prior knowledge.

In addition, it is unclear whether the effect of prior knowledge on different feature types remains with the passage of time. Most prior knowledge-related effects are obtained right after encoding or on the next day. Such effects are consistent with the hypothesis that the assimilation of information consistent with prior knowledge into an existing knowledge system can proceed rapidly (Tse et al., 2007; McClelland, 2013). Recent studies have suggested that sleep facilitates memory consolidation, leading to slower forgetting rate when the information is consistent with prior knowledge (Durrant et al., 2015; Hennies et al., 2016). For example, in a study of Hennies et al. (2016), participants encoded new facts (e.g., Pontu lives for a long time), which were either related or completely unrelated to the established knowledge. The results showed that memory for facts with prior knowledge was enhanced, and the effect of prior knowledge was greater when the test was 24 h later than when tested immediately after encoding. The fact of less decay over time for information related to prior knowledge is important because unlike episodic information, semantic information can be maintained for a life-long period. We continued to explore whether perceptual and functional features were forgotten at a similar rate within a 1-week window.

To summarize, the central question raised in this study is whether prior knowledge enhances memory for different feature types when new information is acquired and maintained over time. To address this issue, we first defined the level of prior knowledge (i.e., high and low) as familiar and unfamiliar categories (DeWitt et al., 2012; Hennies et al., 2016). When participants are familiar about general knowledge in these categories and can generate many exemplars from them, they could use it as the prior knowledge in memory tasks. Prior knowledge provides a semantic context with which to form elaborate or distinctive memories and boost memory performance. The category selection was based on standard norms (Battig and Montague, 1969; Van Overschelde et al., 2004; e.g., four-foot animals as a familiar category, and birds as an unfamiliar category), and the selected categories were confirmed by a separate group of participants. We then selected unfamiliar exemplars from familiar and unfamiliar categories. The sentences were generated for each exemplar (i.e., target word) and included perceptual and functional features. Familiar exemplars were not included because the study aimed to explore mechanisms of acquiring and maintaining new factual information.

In Experiment 1, participants learned all the sentences. Then, after 10-min, 1-day, and 1-week intervals, their memory for the sentences was tested by an old/new recognition test, followed by a remember/know/guess judgment. Recollection and familiarity processes were estimated to examine the cognitive mechanisms underlying the effect of prior knowledge. Different retention intervals were used to examine whether the effect of prior knowledge enhanced recent and remote memories for factual knowledge. We hypothesized that sentences that involve familiar categories are recognized better than those that involve unfamiliar categories, and this pattern should be especially evident for the perceptual features because they are easily assimilated into existing prior knowledge. As facts for new exemplars with prior knowledge can be consolidated quickly (Tse et al., 2007; Moscovitch et al., 2016), we hypothesized that the enhanced effect of prior knowledge would last for recent and remote memories.

In addition to the relationship to prior knowledge, the other difference between the perceptual and functional features is that the former can be easily imagined (see **Supplementary Material**). To explore whether feature type, rather than vividness, modulated the effect of prior knowledge on acquiring new information, we included sentences that described episodes including the exemplars in Experiment 2. Episodic information for events related to objects includes detailed and vivid features (e.g., duration, time, location, consequence, and emotion; Levine et al., 2002; Anderson et al., 2017). However, different from the perceptual features of objects, the episodic features are not associated with prior knowledge. In Experiment 2, participants were asked to learn sentences that included perceptual, functional, and episodic features. During the test, they were asked to recall these features by cues at different retention intervals. We hypothesized that only information associated with prior knowledge is remembered better and retained for a long time. Prior knowledge provides conceptual information to construct semantic representations and boost memory performance for perceptual knowledge but not memory for episodic information.

## Experiment 1

### Materials and Methods

#### Participants

Forty-one participants were recruited in the study. Among them, 23 participants (seven males) with a mean age of 22.30 ± 2.75 years were enrolled in the memory task, and another 18 participants (seven males, with mean age of 21.0 ± 2.0 years) were enrolled in the control task to ensure that the baseline accuracy of sentence judgment was matched related to prior knowledge. All of the participants were native Chinese speakers, and they all gave written informed consent in accordance with the procedures and protocols, which were approved by the Review Board of School of Psychological and Cognitive Sciences, Peking University.

#### Materials

Three within-subject factors were included in the study: level of prior knowledge (familiar category as high prior knowledge, unfamiliar category as low prior knowledge), feature type (perceptual, functional), and retention interval (10 min, 1 day, 1 week).

We first selected 11 familiar (e.g., vegetable) and 12 unfamiliar categories (e.g., insect; **Table 1**). Among them, nine familiar and nine unfamiliar categories were from Battig and Montague (1969) and Van Overschelde et al. (2004). In the study of Van Overschelde et al. (2004), category potency and rank are regarded as the indexes to represent category familiarity. Category potency is computed by dividing the total number of responses given for a category by the total number of participants who responded to that category. The rank score is the mean potency for each category, where the lower the score is, the more familiar the category is. The mean category potency for the familiar and unfamiliar categories were 6.98 ± 1.80 and 5.88 ± 0.83, respectively, and the mean category ranks for the two categories were 21.30 ± 17.37 and 33.44 ± 13.43, respectively. Considering cultural difference and time development, we added two familiar (i.e., Chinese food, Chinese daily utensils) and three unfamiliar categories (i.e., merchant brand, Chinese medicine, and Chinese tea).

**Table 1:** Stimulus category.

High prior knowledge	Exemplar	Low prior knowledge	Exemplar
Fruit	Psidium guajava	Bird	Jackdaw
Vegetable	Chicory	Fish	Plecoglossus altivelis
Four-foot animal	Skunk	Insect	Porcellio
Human organ	Pituitary	Flower	Epipremnum aureum
Natural phenomenon	Hoarfrost	Tree	Cycas revolute
Chinese daily utensil	Ladle	Chinese medicine	Atractylodes
Transportation	Barge	Chinese tea	Cui Luo
Furniture	Tapestry	Dance	Sword dance
Tool	Punch	Merchant brand	Dolby
Sport	Squash	Musical instrument	Xylophone
Chinese food	Zui Xia	Cloth	Acrylic
		Car brand	MAXUS

The familiarity of the 23 categories was also rated by an additional 19 participants (13 males, with a mean age of 22.6 ± 2.58 years). For each category, the participants were asked to rate whether they were familiar of its general knowledge (DeWitt et al., 2012) and whether they could generate many of its exemplars (Battig and Montague, 1969; Van Overschelde et al., 2004; one for most unfamiliar and seven for most familiar). The mean familiarity for familiar and unfamiliar categories were 5.25 ± 0.77 and 3.99 ± 0.73, respectively. The difference was significant, *F*(1,18) = 256.71, *p <* 0.001, η*p*^2^ = 0.93, which confirmed the validity of category selection.

We then selected unfamiliar exemplars as target words within each category. To confirm that the exemplars were unfamiliar and that the familiarity was matched across conditions, the 19 participants were also asked to rate to what extent they were familiar with the features of the exemplars (one for most unfamiliar, and seven for most familiar). The mean exemplar familiarity for familiar and unfamiliar categories were 3.01 ± 0.74 and 2.86 ± 0.53, respectively. The difference was not significant, *t*(18) = 1.50, *p* = 0.31, which confirmed that exemplars are matched in exemplar familiarity, whether the prior knowledge is high or low. In addition, the logarithmic word frequency (Friederici and Frisch, 2000) and the number of strokes for the target words were also matched between familiar and unfamiliar categories (*p*’s > 0.30; **Table 2**).

**Table 2:** Stimulus features related to prior knowledge.

	High PK	Low PK
Category potency	6.98 ± 1.80	5.88 ± 0.83
Category rank	21.30 ± 17.37	33.44 ± 13.43
Category familiarity	5.25 ± 0.77	3.99 ± 0.73
Target word familiarity	3.01 ± 0.74	2.86 ± 0.53
Target word frequency	6.56 ± 0.48	6.75 ± 0.62
Target word strokes	17.39 ± 5.67	17.64 ± 4.64
Keyword length	1.89 ± 0.82	1.79 ± 0.71
Keyword frequency	8.09 ± 1.05	8.11 ± 1.01
Sentence length	35.89 ± 6.45	36.47 ± 6.66
Vividness rating	3.47 ± 1.01	3.32 ± 0.97

*PK refers to prior knowledge.*

The unfamiliar exemplars were used to generate sentences that described their features. Each sentence contained the name of the category the target word belongs to, two perceptual features, and two functional features (**Table 3**). Based on the standard applied in the study of McRae et al. (2005), the perceptual features are defined as information that can be seen or perceived, such as color, shape, and odor, whereas the functional features are defined as information related to perceptual-irrelevant features, such as their usage and location. Each feature description was separated by a comma, and the location of perceptual and functional information within the sentence was counterbalanced across sentences. Each sentence during encoding contained 36.36 ± 4.64 Chinese characters (including punctuation), and the average length for each short sentence during retrieval was 8.87 ± 1.64 characters. The sentence length was not significantly different between familiar and unfamiliar categories, *p* > 0.40 (**Table 3**).

**Table 3:** Sentence examples in Experiments 1 and 2.

**Semantic sentences in Experiments 1 and 2**	
Target words with high prior knowledge	
Study	
The psidium guajava is a species of fruit. The color of its peel is lime-green. It tastes crisp and sweet. It originally grows in America. It can be used to treat diarrhea.	
Test in Experiment 1	Test in Experiment 2
The color of the psidium guajava’s peel is lime-green. (Correct)	The color of the psidium guajava’s peel is ( ).
The psidium guajava originally grows in America. (Correct)	The psidium guajava originally grows in ( ).
The color of the psidium guajava’s peel is red. (Incorrect)	
The psidium guajava originally grows in Asia. (Incorrect)	
Target words with low prior knowledge	
Study	
The cycas revolute is a species of trees. Its leaves are feather-like. The color of its seeds is orange. It blooms at least 10 years after adulthood. It spreads over central China.	
Test in Experiment 1	Test in Experiment 2
The cycas revolute’s leaves are feather-like. (Correct)	The cycas revolute’s leaves are ( )
The color of the cycas revolute’s seeds is orange. (Correct)	The cycas revolute blooms every ( ) years.
The color of the cycas revolute’s seeds is brown. (Incorrect)	
The cycas revolute blooms every 2 years. (Incorrect)	
**Episodic sentences in Experiment 2**	
Study	
Yesterday morning, I bought three pounds of psidium guajavas that were planted in Shandong province from the fruit store in front of the Summer Palace.	
Last week, I saw the blossom of three flowers on the cycas revolute next to the news stall when I went to school.	
Test	
Yesterday morning, I bought ( ) pounds of psidium guajavas that were planted in Shandong province from the fruit store in front of ( ).	
Last week, I saw the blossom of three flowers on the cycas revolute next to the ( ) when I went to ( ).	

To test for memory of the perceptual and functional features, we also generated incorrect descriptions for features with similar aspects (e.g., red vs. yellow in color). Thus, each target word had a total of eight short sentences (four correct and four incorrect; **Table 3**). The mean length of the perceptual and functional short sentences were 8.56 ± 1.65 and 9.20 ± 1.63 characters, respectively (**Table 4**). The functional sentences were longer than the perceptual sentences, *F*(1,284) = 11.17, *p <* 0.001, η*p*^2^ = 0.04. This was partly due to the fact that in Chinese, the perceptual features could be described by one or two characters, but the functional features are described by over two characters. Importantly, there was no significant effect of prior knowledge and the interaction between level of prior knowledge and feature type (*F*’s < 2, *p*’s > 0.20).

**Table 4:** Stimulus features related to feature types.

	Perceptual		Functional		Episodic	
	High PK	Low PK	High PK	Low PK	High PK	Low PK
Keyword length	1.78 ± 0.74	1.81 ± 0.78	2.00 ± 0.90	1.78 ± 0.65	1.97 ± 0.69	2.08 ± 0.60
Keyword frequency	8.04 ± 1.07	8.04 ± 1.08	8.04 ± 1.09	8.28 ± 0.91	7.91 ± 0.86	7.64 ± 0.95
Sentence length	8.40 ± 1.65	8.72 ± 1.64	9.21 ± 1.58	9.21 ± 1.70	34.25 ± 6.03	35.39 ± 6.11
Vividness rating	3.86 ± 0.97	3.08 ± 0.91	3.68 ± 0.92	2.95 ± 0.90	5.07 ± 1.21	5.16 ± 1.14

*PK refers to prior knowledge.*

We also examined whether features of keywords (e.g., word frequency and word length) were matched across conditions. The keywords referred to the words that denoted perceptual and functional features and were replaced with incorrect words in the incorrect sentences. The mean logarithmic word frequency (Friederici and Frisch, 2000) for familiar and unfamiliar categories were 8.09 ± 1.05 and 8.11 ± 1.01, and the mean word length were 1.89 ± 0.82 characters and 1.79 ± 0.71 characters, respectively (**Table 2**). There were no significant effects of prior knowledge, feature type and the interaction (*F*’s < 2, *p*’s > 0.10) for the two keyword features, suggesting that they are matched across conditions (**Table 4**).

A total of 72 sentences were randomly divided into three sets, each with 24 sentences, half of which described exemplars from the familiar categories, whereas the other half described exemplars from the unfamiliar categories. The three sets were used as material for three time intervals. Each sentence was divided into four short sentences during retrieval (96 total for each interval), half of which described the perceptual features, whereas the other half described the functional features. Thus, each condition (e.g., perceptual features for familiar category at 10 min) had 96 short sentences for analysis, half of the short sentences were correct and half were incorrect. The three sets had no significant differences in average baseline accuracy and various lexical-semantic features, such as exemplar familiarity, frequency and number of strokes for the target words, frequency and word length of the keywords, and sentence length (*p*’s > 0.20). The sets were counterbalanced, and thus, each set had an equal opportunity of being used at different retention intervals.

#### Procedure

**FIGURE 1:**
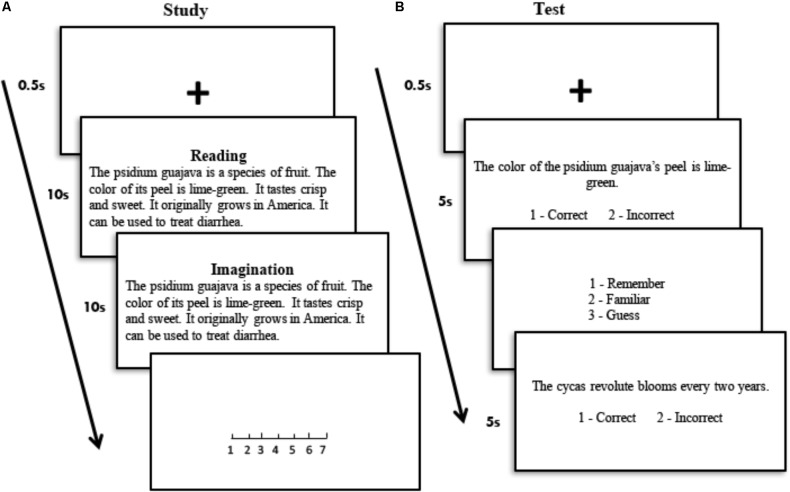
Procedure of the study and test session in Experiment 1. **(A)** During the study phase, participants were presented with the sentence to describe each unfamiliar exemplar, and were asked to read and imagine it. **(B)** During the test phase, the participants were asked to make an old/new judgment and a remember/know/guess judgment (if the old judgment was made). The Chinese sentences were translated into English for illustration purpose.

For the participants who were recruited in the formal study, they learned all of the sentences in the same day; then, they performed the recognition tests at intervals of 10 min, 1 day, and 1 week. During the study phase, the participants were presented with each of the 72 sentences for 10 s, during which they read the sentence silently (**Figure 1A**). Then, the same sentence was presented for another 10 s, during which they imagined the sentence as vividly as possible, and judged the level of vividness (range from one as least vivid to seven as most vivid). Within each sentence, the category description (e.g., a target word is an animal) was presented at first for all the participants, followed by the descriptions for two perceptual and two functional features. Half of the participants were shown the two perceptual descriptions, followed by the two functional descriptions, and the other half of the participants were in the opposite. The order of the perceptual and functional descriptions within each sentence was counterbalanced across the participants.

During the test phase, the sentence for each target word was divided into four short sentences (**Table 3**). The category sentences were not presented. Each of the short sentences and the corresponding incorrect sentences (a total of 192 for each time interval, 96 correct and 96 incorrect) was presented for 5 s, and the participants were asked to judge whether the description was correct (**Figure 1B**). If the sentence was judged to be correct, it was presented again and the participants were asked to judge whether they remembered, knew, or guessed it. If the participants judged that they could retrieve sentence-related details or contexts, they responded with a judgment of “remember”; if they only felt that the sentence was familiar without any detailed information, they responded with a judgment of “know.” If they did not believe that they retrieved the sentence by the above two processes, they responded with a judgment of “guess.” The short sentences were pseudo-randomly presented at each time interval for each participant so that no more than three sentences for each condition were presented consecutively. The order of the correct and incorrect descriptions for each feature was counterbalanced so that half of the correct descriptions were presented first in each condition. The press button for the recognition judgment was counterbalanced across the participants.

Before each test phase, to avoid a rehearsal from the study phase, the participants were asked to count backward by seven continuously from 1000 for 5 min. The participants had separate opportunities to practice study and test trials before the formal phases.

For the participants who were enrolled in the control task, during the task, they were presented 576 (288 correct, 288 incorrect) short sentences for the 72 unfamiliar words without encoding phase, and asked to judge whether the description was correct or not. The sentences included four short descriptions for each word’s perceptual and four functional features, half were correct and half incorrect. The short sentences were presented in a pseudo-random order, and no more than three short sentences from the same target word or the same condition were presented continuously. The order of the correct and incorrect descriptions for each feature was counterbalanced so that half of the correct descriptions were presented first. The press button for the judgment was counterbalanced across the participants.

#### Data Analysis

The Hit rate, FA rate, and corrected recognition (Hit–FA) for the recognition task were calculated and analyzed separately using repeated ANOVA measures with level of prior knowledge (high, low), feature type (perceptual, functional), and retention interval (10 min, 1 day, 1 week) as within-subject factors. The forgetting rate was estimated by the interaction between the retention interval and other factors (Slamecka and McElree, 1983; Gardiner and Java, 1991; Hockley and Consoli, 1999). Two of the participants’ data were excluded due to low memory accuracy (>2SD). We also performed the results for all the participants, and excluding the two subjects’ data did not impact the pattern of statistical results. Partial eta squared (η*p*^2^) was calculated to estimate the effect size of each analysis. *Post hoc* pairwise comparisons were Bonferroni-corrected (*p* < 0.05, two tailed).

The recollection and familiarity processes were estimated using the independent K (IRK) procedure (Yonelinas, 2002), in which R responses are assumed to estimate recollection, whereas familiarity is estimated as the proportion of K responses divided by the proportion of non-R responses. According to this procedure, the R and K responses are not only mutually exclusive, but they are also independently estimated. Then, the R and IRK responses were corrected using FA: recollection = *p*(R, hit) – *p*(R, FA); familiarity = *p*(K, hit)/(1 − *p*(R, hit)) − *p*(K,FA)/(1 – *p*(R,FA)). Repeated measures ANOVA tests were performed separately for the recollection and familiarity processes with the retention interval, category familiarity, and features as within-subject factors.

### Results

During the encoding task, the participants rated sentences with high prior knowledge as being more vividly than sentences with low prior knowledge [5.01 ± 0.52 and 4.67 ± 0.57, *F*(1,20) = 22.50, *p <* 0.001, η*p*^2^ = 0.51].

**FIGURE 2:**
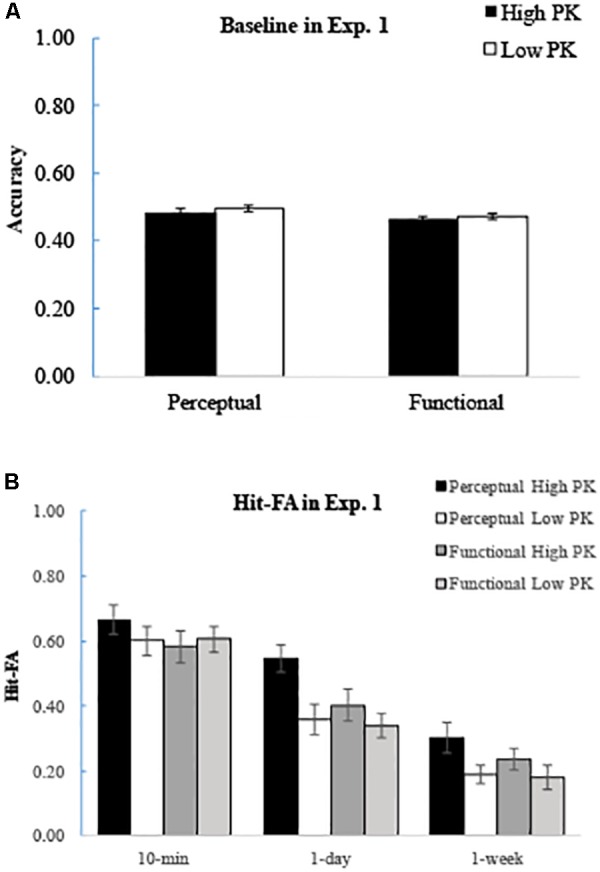
Results of Experiment 1. **(A)** Baseline accuracy of recognition in each condition. Both perceptual and functional features were recognized at chance level. **(B)** Corrected recognition for perceptual and functional details at each retention interval. Prior knowledge enhanced memory for perceptual features but not for functional features, and the effects occurred for up to 1 week. The error bars represent the standard errors of the means. The abbreviation of PK refers to prior knowledge.

For the control group, the average accuracy was 0.48 ± 0.03 (**Figure 2A**). The ANOVA with prior knowledge and feature type as factors showed that there were no significant effects of prior knowledge [*F*(1,17) = 1.33, *p* = 0.26, η*p*^2^ = 0.07), feature type [*F*(1,17) = 3.78, *p* = 0.07, η*p*^2^ = 0.07. Mean: 0.49 ± 0.03 for perceptual and 0.47 ± 0.03 for functional descriptions], or their interaction [*F*(1,17) = 0.08, *p* = 0.78, η*p*^2^ = 0.005]. The accuracy was not significantly different from the chance level (0.5) for each condition (*p*’s > 0.20).

For the corrected recognition, ANOVA of prior knowledge ^∗^ feature type ^∗^ retention interval showed that the sentences with high prior knowledge were recognized better than those with low prior knowledge [*F*(1,20) = 20.24, *p <* 0.001, η*p*^2^ = 0.50], and the perceptual features were recognized better than the functional features [*F*(1,20) = 7.20, *p* = 0.01, η*p*^2^ = 0.19]. There was a significant interaction between prior knowledge and feature type [*F*(1,20) = 16.28, *p* = 0.001, η*p*^2^ = 0.45]. Further analysis showed that enhanced memory performance for prior knowledge only appeared for the perceptual features (*p* < 0.001), but not for the functional features (*p* = 0.12; **Figure 2B**). Memory performance decreased significantly over time [*F*(2,40) = 129.03, *p* < 0.001, η*p*^2^ = 0.87], and there was a marginal significant interaction between prior knowledge and retention interval [*F*(2,40) = 3.10, *p* = 0.056, η*p*^2^ = 0.13]. Further analysis showed that the effect of prior knowledge occurred at 1-day and 1-week intervals (*p*’s < 0.02) but not at 10 min (*p* = 0.45). The results suggested that the information with higher prior knowledge remains for a longer time.

For the Hit rate, there was a significant interaction between prior knowledge and feature type [*F*(1,20) = 8.45, *p* = 0.01, η*p*^2^ = 0.30], as the effect of prior knowledge for the Hit rate only appeared for the perceptual features (*p* < 0.001), but not for the functional features (*p* = 0.83). The accuracy decreased significantly over time [*F*(2,40) = 26.29, *p* < 0.001, η*p*^2^ = 0.57], but no significant interactions related to retention interval were found (*p*’s > 0.1; **Table 5**).

**Table 5:** Results of Experiment 1.

		Perceptual		Functional	
		High PK	Low PK	High PK	Low PK
Hit	10 min	0.87 ± 0.14	0.84 ± 0.13	0.84 ± 0.14	0.83 ± 0.12
	1 day	0.84 ± 0.10	0.81 ± 0.15	0.77 ± 0.14	0.78 ± 0.14
	1 week	0.74 ± 0.13	0.70 ± 0.17	0.70 ± 0.18	0.70 ± 0.19
FA	10 min	0.23 ± 0.14	0.26 ± 0.17	0.26 ± 0.19	0.26 ± 0.17
	1 day	0.28 ± 0.23	0.47 ± 0.21	0.40 ± 0.22	0.47 ± 0.22
	1 week	0.50 ± 0.19	0.58 ± 0.21	0.50 ± 0.18	0.54 ± 0.17
Recollection	10 min	0.51 ± 0.25	0.46 ± 0.25	0.49 ± 0.27	0.46 ± 0.25
	1 day	0.37 ± 0.22	0.29 ± 0.19	0.30 ± 0.19	0.27 ± 0.19
	1 week	0.18 ± 0.17	0.14 ± 0.13	0.12 ± 0.19	0.12 ± 0.14
Familiarity	10 min	0.26 ± 0.25	0.22 ± 0.18	0.16 ± 0.25	0.23 ± 0.21
	1 day	0.18 ± 0.21	0.18 ± 0.19	0.18 ± 0.20	0.14 ± 0.17
	1 week	0.12 ± 0.18	0.13 ± 0.12	0.08 ± 0.11	0.10 ± 0.13

For the FA rate, the results showed that the sentences with high prior knowledge had a lower FA rate than those with low prior knowledge [*F*(1,20) = 17.21, *p* < 0.001, η*p*^2^ = 0.47]. The FA rate increased significantly over time [*F*(2,40) = 51.04, *p* < 0.001, η*p*^2^ = 0.72; **Table 5**]. There was a significant interaction between prior knowledge and feature type [*F*(1,20) = 5.30, *p* = 0.03, η*p*^2^ = 0.21]. Further analysis showed that the effect of prior knowledge was larger for the perceptual features (*p* < 0.001) than the functional features (*p* = 0.06). In addition, there was a significant interaction between prior knowledge and retention interval [*F*(2,40) = 3.75, *p* = 0.03, η*p*^2^ = 0.16]. Further analysis showed that the effect of prior knowledge occurred at 1-day and 1-week intervals (*p*’s < 0.05) but not at 10 min (*p* = 0.22). It suggested that the effect of prior knowledge at longer interval is mainly due to lower FA for the information with high prior knowledge. Thus, the prior knowledge facilitates the memory by increasing the ability to distinguish the information from the interferences.

With regard to the recollection estimates, the results showed that the sentences with high prior knowledge had more recollection contributions than those with low prior knowledge [*F*(1,20) = 8.98, *p* < 0.01, η*p*^2^ = 0.31], and the perceptual features had more recollection involvement than the functional features [*F*(1,20) = 4.28, *p* = 0.05, η*p*^2^ = 0.18]. Similar to the results of the corrected recognition, there was a significant interaction between the prior knowledge and feature type [*F*(1,20) = 4.60, *p* = 0.04, η*p*^2^ = 0.19], showing that the enhanced effect of prior knowledge only appeared for the perceptual features (*p* = 0.001) but not for the functional features (*p* = 0.21; **Table 5**). The recollection contribution decreased significantly over time [*F*(2,40) = 40.36, *p* < 0.001, η*p*^2^ = 0.67], but no significant interactions related to retention interval were found (*p*’s > 0.1). This suggested that prior knowledge enhances memory for the perceptual features through the recollection process.

The familiarity contribution decreased over time [*F*(2,40) = 5.55, *p* = 0.01, η*p*^2^ = 0.22; **Table 5**], and the perceptual features had a greater familiarity contribution than the functional features [*F*(1,20) = 5.21, *p* = 0.03, η*p*^2^ = 0.21]; however, no other significant effects appeared (*F*’s < 2, *p*’s > 0.5). We also compared the contribution of recollection and familiarity at different retention intervals, and the results showed that the contribution of recollection was higher than that of familiarity at 10-min (*p*’s < 0.001) and 1-day intervals (*p*’s < 0.03), but they were comparable at 1-week intervals (*p*’s > 0.20).

### Discussion

The results of Experiment 1 showed a significant interaction between prior knowledge and feature type for the corrected recognition. Prior knowledge enhanced memory for the perceptual features but not for the functional features, and the effect occurred for up to 1 week. This effect was due to a higher Hit rate and lower FA rate for the sentences with higher (vs. lower) prior knowledge. The effect of prior knowledge on the perceptual features was mainly driven by the recollection process. This suggested that prior knowledge facilitate memory for new information related to perceptual features. Once memory is enhanced, the effect of prior knowledge stabilizes for recent and remote memories. In addition, the results of Experiment 1 showed a significant interaction between prior knowledge and retention interval. For the corrected recognition and FA rate, the effect of prior knowledge occurred at 1-day and 1-week intervals. This finding suggested that prior knowledge leads to a slower forgetting rate, which was consistent with previous findings (Durrant et al., 2015; Hennies et al., 2016).

The aim of Experiment 2 was to explore whether feature type, rather than encoding vividness, modulated the effect of prior knowledge on acquiring new information. As shown in **Supplementary Material** and **Table 4**, in the vividness rating, the episodic sentences had the highest vividness scores, then the perceptual sentences, and the functional sentences had the lowest vividness scores. In addition to the sentences used in Experiment 1, we included sentences that described episodic events related to the exemplars. Participants learned these sentences and were asked to perform a cued recall task at different retention intervals. Previous studies have suggested that the effect of prior knowledge is more evident in recall tasks than in recognition (Alba and Hasher, 1983; Long and Prat, 2002). We showed that the effect of prior knowledge was mainly due to the recollection contribution in Experiment 1. Therefore, we hypothesized that the effect of prior knowledge exists in the cued-recall task, especially for perceptual features. In addition, as episodic features are not essential to obtain the conceptual knowledge required for a certain unfamiliar stimulus, we hypothesized that memory for episodic features does not demonstrate an enhanced effect of prior knowledge, although the episodic features are more vividly than the perceptual and functional features.

## Experiment 2

### Materials and Methods

#### Participants

Forty-six participants were recruited in the study. Among them, 25 participants (nine males) with a mean age of 22.40 ± 2.27 years were enrolled in the memory task, and another 21 participants (five males, with a mean age of 22.67 ± 1.53 years) were enrolled in the control task to ensure that the baseline accuracy of filling in the fragment sentences was matched related to the level of prior knowledge. All of the participants were native Chinese speakers, and they all gave written informed consent in accordance with the procedures and protocols, which were approved by the Review Board of School of Psychological and Cognitive Sciences, Peking University.

#### Materials

Three within-subject factors were included in the study: level of prior knowledge (high, low), feature type (perceptual, functional, episodic), and retention interval (10 min, 1 day, 1 week). There were two types of sentences for each target word; one included the perceptual and functional features, whereas the other included episodic features. The 72 sentences for the perceptual and functional features were the same as those used in Experiment 1 (i.e., semantic sentences). The 72 episodic sentences for the same 72 target words were created separately, each with different features, including time, location, human, and other episodic information. The perceptual and functional features were not used for the episodic sentences. Thus, the features did not overlap for semantic and episodic sentences. Each episodic sentence described an event related to a target word, which was a piece of detailed information in the sentence (**Table 3**). Within each sentence, the information of time and human subject (i.e., I, we) was presented at first, Other information was then provided to organize a unitized episode. The position of the target word was determined by episodes. Each episodic sentence during encoding contained 34.82 ± 6.05 Chinese characters (including punctuation), with no significant difference for the level of prior knowledge (*p* > 0.80; **Table 4**). No significant difference was found between the length of semantic and episodic sentences (*p* > 0.10).

We also examined whether features of the keywords (e.g., word frequency and word length) were matched across conditions. The keywords for the episodic sentences referred to the words that denoted episodic details and were unfilled in the recall task. The mean logarithmic keyword frequency (Friederici and Frisch, 2000) for familiar and unfamiliar categories were 7.91 ± 0.86 and 7.64 ± 0.95, and the mean keyword length were 1.97 ± 0.69 characters and 2.08 ± 0.60 characters, respectively (**Table 4**). The effects of prior knowledge and the interactions between prior knowledge and feature type were not significant for both keyword frequency and length (*F*’s < 2, *p*’s > 0.10). There were significant effects of feature type for the keyword frequency [*F*(2,426) = 6.96, *p* = 0.001, η*p*^2^ = 0.03] and keyword length [*F*(2,426) = 3.76, *p* = 0.02, η*p*^2^ = 0.02]. Further analysis showed that the episodic sentences had lower keyword frequency than the functional sentences (*p* = 0.001), and had longer keyword length than the perceptual sentences (*p* = 0.01). No other significant differences were found (*p*’s > 0.30).

The sentences for the 72 exemplars were randomly divided into three sets, each with semantic and episodic sentences, half of which included words from familiar categories, whereas the other half included words from unfamiliar categories. The three sets were used as materials for three retention intervals. These sets had no significant in average baseline accuracy and various lexical-semantic features, such as exemplar familiarity, frequency and number of strokes for the target word, frequency and word length of the keywords, and sentence lengths (*F’*s < 1, *p*’s > 0.50). The sets were counterbalanced across the participants, and thus, each set had an equal chance of being used under different conditions.

#### Procedure

For the participants who were recruited in the formal study, they learned all of the sentences in the same day; then, they performed the recall tests at intervals of 10 min, 1 day, and 1 week. The study phase for each trial was the same as that of Experiment 1, except that each of the episodic sentences was presented for 20 s after the semantic sentence for the same target word was read, imagined, and rated. This procedure ensured that the participants had the opportunity to obtain semantic information for the unfamiliar words before processing the episodic sentences. During each test phase, the sentences for each target word were divided into semantic and episodic parts, with some features unfilled. These sentences were pseudo-randomly presented, and the participants were asked to recall detailed information and speak aloud the whole sentences in a self-paced speed. The unfamiliar words were constantly presented in fragment sentences. The participants’ responses were recorded and analyzed. The order of the perceptual and functional features in semantic sentences was counterbalanced within the participants. The descriptions of the semantic and episodic parts (a total of 144 for each time interval) were pseudo-randomly presented at each retention interval for each participant so that no more than three descriptions in the same condition were presented consecutively.

For the control group, the 72 semantic sentences and 72 episodic sentences were presented in fragments (one semantic and one episodic for each word). The unfilled parts (i.e., keywords) were perceptual and functional features in semantic sentences, and contextual information in episodic sentences. The participants were asked to speak aloud the whole sentence by using their existing knowledge or by guessing when the prior knowledge was unavailable. Their responses were recorded and the accuracy of each sentence was calculated and analyzed afterward. The sentences were presented in a pseudo-random order, and no more than three sentences from the same condition were presented consecutively. The order of the perceptual and functional features in the semantic sentences was counterbalanced within the participants.

#### Data Analysis

The recall accuracy each condition was calculated. The repeated measures ANOVA was performed with level of prior knowledge, feature type (perceptual, functional, episodic), and the retention interval (10 min, 1 day, 1 week) as within-subject factors. One subject’s data were excluded due to low memory accuracy (>2SD). The recall baseline for each condition was also analyzed with prior knowledge (familiar, unfamiliar) and feature type (perceptual, functional, episodic) as within-subject factors. Other parts of data analysis were the same as that in Experiment 1.

### Results

During the encoding task, the participants rated episodic information as more vivid than semantic information [5.00 ± 0.84 and 4.18 ± 0.92, *F*(1,23) = 16.02, *p* < 0.001, η*p*^2^ = 0.58]. A significant interaction was found between sentence (semantic, episodic) and prior knowledge [*F*(1,23) = 21.28, *p* < 0.001, η*p*^2^ = 0.48]; however, the effect of prior knowledge was significant for the episodic information (*p* < 0.01) and semantic features (*p* < 0.05).

**FIGURE 3:**
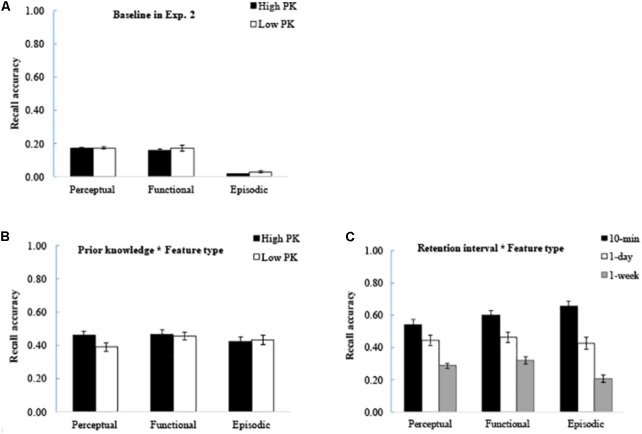
Results of Experiment 2. **(A)** Baseline accuracy of recall in each condition. **(B)** Interaction between prior knowledge and feature type. The enhanced memory due to prior knowledge appeared for perceptual features, but not for functional and episodic features. **(C)** Interaction between retention interval and feature type. The memory for episodic features was forgotten more rapidly than that for semantic features. The error bars represent the standard errors of the means. The abbreviation of PK refers to prior knowledge.

For the recall baseline, the ANOVA results showed that there was a significant effect of feature type [0.17 ± 0.03 for perceptual and 0.16 ± 0.06 for functional, and 0.02 ± 0.02 for episodic feature. *F*(2,36) = 96.98, *p* < 0.001, η*p*^2^ = 0.85]. Further analysis showed that perceptual and functional features were filled more accurately than episodic features (*p*’s < 0.001), but no significant difference was found between the perceptual and functional features (*p* = 1.00). The effect of prior knowledge [0.11 ± 0.04 for familiar and 0.12 ± 0.05 for unfamiliar. *F*(1,18) = 2.27, *p* = 0.10, η*p*^2^ = 0.10] and the interaction between prior knowledge and feature type [*F*(2,36) = 0.22, *p* = 0.80, η*p*^2^ = 0.80] were not significant (**Figure 3A**). These results suggested that the conceptual knowledge of the features described in the sentences are optimally matched whether the level of prior knowledge is high or low.

During the recall task, the results showed that the sentences with high prior knowledge were recalled better than those with low prior knowledge [*F*(1,23) = 5.23, *p* = 0.03, η*p*^2^ = 0.19]. Furthermore, a significant interaction was found between prior knowledge and feature type [*F*(2,46) = 6.82, *p* = 0.003, η*p*^2^ = 0.23], which was consistent with that in Experiment 1. Further analysis indicated that the effect of prior knowledge only appeared for the perceptual features (*p* < 0.001) but not for the functional (*p* = 0.58) and episodic (*p* = 0.46) features (**Figure 3B**). The results suggested that prior knowledge enhances memory for the perceptual features but not for the functional features, which was similar to that in Experiment 1. In addition, prior knowledge failed to enhance memory for the episodic features.

Moreover, a significant interaction was found between feature type and retention interval [*F*(4,92) = 13.34, *p* < 0.001, η*p*^2^ = 0.37]. Further analysis showed that at 10 min, the memory for episodic features was higher than that for perceptual and functional features (*p*’s < 0.05). The memory for the three feature types were comparable at 1-day interval (*p*’s > 0.50). At 1-week interval, the memory for episodic features was lower than that for perceptual and functional features (*p*’s < 0.001). This suggested that memory for the episodic features is forgotten more rapidly than that for the semantic features (**Figure 3C**).

### Discussion

The results of Experiment 2 showed a significant interaction between prior knowledge and feature type in the cue-recall task, given that the enhanced memory performance due to prior knowledge appeared for the perceptual features but not for the functional and episodic features. The findings were obtained when the baseline performance was well controlled. The results suggested that the effect of prior knowledge is modulated by feature type but not by vividness. Although the perceptual and episodic features were vivid and easily imagined, only the former showed enhanced memory when the prior knowledge was available.

In addition, when semantic and episodic features were compared, memory for the episodic features was forgotten more rapidly than that for the semantic features. Thus, prior knowledge may enhance memory consolidation for relevant information easily assimilated into the existing knowledge system, leading to slower forgetting rate for new factual information.

## General Discussion

In this study, factors of prior knowledge, feature type, and retention interval were manipulated to explore their effects on memory for new factual information. We asked whether prior knowledge enhances memory for different feature types when new information is acquired and maintained over time. There were two main findings. First, prior knowledge enhanced memory for the perceptual features but not for the functional and episodic information. This enhancement depended on the recollection process. Second, a significant interaction was found between prior knowledge and retention interval, showing that prior knowledge slowed the forgetting rate for newly learned factual information. But the effect of prior knowledge on the perceptual features remained stable over time. This study highlighted the importance of prior knowledge in acquiring new factual information with the passage of time.

### Perceptual Features and Prior Knowledge

The novel finding of the study was that prior knowledge enhanced memory for the perceptual features, but not for the functional features. Although previous studies have reported that prior knowledge enhanced memory for relevant information (Long and Prat, 2002; DeWitt et al., 2012; van Kesteren et al., 2014; Brod et al., 2016; Hennies et al., 2016), whether its effect is related to knowledge features remains unknown. The results suggested that prior knowledge interacts with the feature type to influence memory of new factual information.

The results supported our hypothesis that the perceptual features are more associated with prior knowledge, and consequently, are more easily assimilated into an existing knowledge system than the functional features. Perceptual features are intrinsic to objects and include information such as color, taste, smell, and surface properties (Warrington and Shallice, 1984; Cree and McRae, 2003; Canessa et al., 2008). By contrast, functional features are extrinsic to objects (Barr and Caplan, 1987) and are part of contexts in which objects are encountered (e.g., how or where a hammer is used). Thus, functional features are not intertwined or well organized within prior knowledge. When prior knowledge is activated, perceptual, rather than functional features, are easily connected with features for other items in the prior knowledge system, which renders organizational processing (Rawson and Van Overschelde, 2008; Rawson et al., 2008) in the brain (Gainotti, 2011). Gainotti (2011) found that rostral parts of the ventral pathway are involved in the subtle and detailed analyses of visual properties of objects, integrating visual data with other perceptual information. The prior knowledge helps the participants distinguish new information from similar information within the existing knowledge system, leading to strong memory representations. The higher Hit rate and lower FA rate for the perceptual (vs. functional) features supported this view. Prior knowledge provides a semantic context with which to form elaborate or distinctive memories and distinguish detailed information from its distractors.

The enhanced effect of prior knowledge for the perceptual features was mainly driven by the recollection process. Similar effects appeared when the recall task was used in Experiment 2. Previous studies have also shown that the effect of prior knowledge is recollection based (Long and Prat, 2002; Brandt et al., 2005; Toth et al., 2011). This finding suggested that prior knowledge increases the availability of details that can support later recollection. During encoding, the participants processed sufficient pieces of detailed information for the perceptual features, as prior knowledge can free attentional resources and allocate them to encode feature details associated with the prior knowledge (DeWitt et al., 2012). During retrieval, the participants used information in the prior knowledge system to aid memory by retrieving information and associations made during encoding. By contrast, as the functional features are more connected with contextual information, encoding them requires hippocampus involvement (Blumenthal et al., 2017). The involvement of the hippocampus inhibits processing information related to prior knowledge (van Kesteren et al., 2012).

The enhanced effect of prior knowledge for the perceptual features was not due to different encoding vividness. In Experiment 2, although encoding episodic sentences led to higher vividness scores, no significant effect of prior knowledge was found in the memory for episodic sentences. Unlike memory for semantic features, memory for episodic features emphasizes the recollection of events from specific time and places (Tulving and Markowitsch, 1998). However, the episodic features are not essential to the knowledge system construction. Therefore, although they may attract attention (Brewer and Lichtenstein, 1975), they are unlikely to be integrated into the existing prior knowledge system, which leads to the absence of the effect of prior knowledge.

In general, participants may first obtain a certain knowledge system before they manifest the effect of prior knowledge for episodic memory (Tulving and Markowitsch, 1998). Studies have suggested that semantic knowledge is a prerequisite for episodic memory (Tulving and Markowitsch, 1998). Semantic knowledge facilitates the acquisition of new episodic memories and the retrieval of information from the semantic system (Greenberg and Verfaellie, 2010). It is possible that the effect of prior knowledge appears for episodic memory when exemplar is familiar to the participants. Further studies could address this issue by including sentences that contain familiar words.

In both experiments, we analyzed detailed information for materials, including the frequency and number of strokes for the target word, frequency and word length of the keywords, and sentence length in each condition. These characteristics were all matched in the level of prior knowledge and counterbalanced across retention interval. They were also mostly matched in feature type in both experiments, except that the perceptual sentences were shorter in length than the functional sentences in Experiment 1; the episodic keywords were longer than the perceptual keywords, and their frequencies were lower than the keywords in functional sentences in Experiment 2. These were mainly due to the fact that in Chinese, describing perceptual features requires fewer words than functional and episodic features. Nevertheless, we consider that the difference in sentence length and keyword features should not influence the memory performance for the following three reasons. First, there were no significant effects of prior knowledge and the interaction related to prior knowledge for these lexical-semantic features. Second, the control accuracy was optimally matched, with chance level and comparable for different levels of prior knowledge and feature types in both experiments. Third, the effect sizes of significant effects of feature type were small for the keywords and sentences (partial η^2^ < 0.05), which suggested that the significant difference is mainly due to large sample sizes (i.e., number of keywords and number of sentences).

### Memory Forgetting and Prior Knowledge

The effect of prior knowledge on different feature types was not influenced by the retention interval. We found that the sentences for the perceptual features with high prior knowledge were remembered better at different retention intervals in recognition and cued recall tasks. Thus, although the general memory performance decreased over time, the enhanced memory due to prior knowledge on the perceptual features remained up to 1 week. We considered that the prior knowledge modulates the encoding process in addition to consolidation. Previous studies have reported that information with prior knowledge is acquired quickly even right after the study (e.g., Brandt et al., 2005; Long and Prat, 2002). Semantic congruence accelerates the onset of neural signals of successful memory encoding (Packard et al., 2017). Once new factual information is acquired, it is consolidated quickly and is retained for a long time. As the perceptual features are associated with information within prior knowledge, semantic networks stabilizes over time for the perceptual features.

On the other hand, prior knowledge slowed the forgetting rate for newly learned factual information in general. New information remained for a longer time when the level of prior knowledge was high (vs. low) in Experiment 1. This result was consistent with that of previous studies in which the effect of prior knowledge was evident at longer intervals, especially after sleep (Durrant et al., 2015; Hennies et al., 2016; Groch et al., 2017; van der Linden et al., 2017). At the same time, the hippocampus is less involved after 24 h than right after encoding (van der Linden et al., 2017). These studies emphasize the role of sleep in consolidating memory related to prior knowledge.

Another interesting finding was that the forgetting rate of semantic and episodic features was different. At 10 min, the memory for episodic features was higher than that for perceptual and functional features. However, at the 1-week interval, the pattern was opposite. This finding suggested that memory for semantic features is forgotten more slowly than that for episodic features. Remote episodic memories are characterized by less detailed information than recently acquired memories (Moscovitch et al., 2016). Differently, memory for conceptual knowledge could be retained for a long time, which accounts for how the detailed conceptual knowledge is acquired and maintained over time.

### Future Directions

The study has potential directions for future investigations. First, some lexical-semantic features were not matched across the feature type. It would be necessary to match these features in future studies to clarify whether prior knowledge facilitates different types of features. Second, it is interesting to distinguish conceptual knowledge as living and non-living categories (Caramazza and Shelton, 1998; Mahon and Caramazza, 2009), and explore whether prior knowledge differentially enhances memory for them (Sartori and Lombardi, 2004; Morrow and Duffy, 2005; Nairne et al., 2017). Previous studies have indicated that living concepts have more features and lower relevance than those related to non-living concepts (Cree and McRae, 2003; Sartori and Lombardi, 2004). Sensory and functional features are differentially weighted for living and non-living categories, in which the former are important for determining living things, whereas the latter for non-living things (Barr and Caplan, 1987; Warrington and McCarthy, 1987; Martin, 2007). Although we included the factor of animacy in the ANOVA analysis in both experiments (see **Supplementary Material**), our study was not designed to explore the factor of animacy, thus the living and non-living categories were not matched across conditions. Further studies are needed to distinguish the effect of feature type and category type on memory performance by carefully manipulating the perceptual and functional features in living and non-living categories. Third, functional features are difficult to specify (Martin and Chao, 2001; Cree and McRae, 2003; Canessa et al., 2008). Whether memory for specific functional features, such as how they are used, is enhanced due to prior knowledge requires further investigation.

## Conclusion

Prior knowledge enhanced memory for the perceptual features but not for the functional and episodic features when new factual information was learned. Such enhancement depended on the recollection process. In addition, the effect of prior knowledge on the perceptual features remained stable over time. This study clarified how different types of new conceptual knowledge were acquired and maintained and highlighted the importance of prior knowledge in acquiring new factual information. It also provided a potential mechanism to account for the fast mapping based concept learning (e.g., Coutanche and Thompson-Schill, 2015).

The way in which memory is formed and consolidated is a central question in the memory field. Studying the influence of prior knowledge on different semantic feature types is of great significance in clarifying how prior knowledge interacts with feature type to modulate memory formation and consolidation (Lewis and Durrant, 2011; Moscovitch et al., 2016). Our results suggested that only information related to prior knowledge could be assimilated into existing knowledge systems and remembered well. Prior knowledge helps assimilate new related information and facilitate the distinction between similar information. This factor makes the encoding and consolidation of relevant information occur rapidly, and once memory trace is stable, the effect of prior knowledge remains for a long period for newly learned factual information.

## Ethics Statement

The study was approved by the ethics committee of School of Psychological and Cognitive Sciences, Peking University. Participants received written and oral information of the study before they gave their written consent.

## Author Contributions

HC designed and performed the research, and analyzed the data. XN designed and performed the research. LW analyzed the data. JY designed the research, analyzed the data, and wrote the paper.

## Conflict of Interest Statement

The authors declare that the research was conducted in the absence of any commercial or financial relationships that could be construed as a potential conflict of interest.
