# Supplementary material for: Acquiring New Factual Information: Effect of Prior Knowledge
Source: Front Psychol. 2018 Sep 24;9:1734. doi: 10.3389/fpsyg.2018.01734 (PMC6165899; doi:10.3389/fpsyg.2018.01734)
Supplement: Supplementary file 1 [file Table_1.docx]

**Vividness rating**

18 participants (8 males, with a mean age of 22.28 ± 2.22 yrs) were enrolled in the vividness rating task to clarify whether the vividness was different for sentences in feature types. All of the participants were native Chinese speakers, and they all gave written informed consent in accordance with the procedures and protocols, which were approved by the Review Board of School of Psychological and Cognitive Sciences, Peking University.

During the vividness rating task, the participants were asked to imagine each short sentence, then they were asked to rate the vividness level for the imagination. The perceptual and functional sentences were presented for 5s, and the episodic sentences were presented for 10 s. This was because the episodic sentences were twice as long as the perceptual and functional sentences, and the sentence length was comparable for the perceptual and functional sentences.

Figure 1. Vividness rating for sentences with different feature types

The results showed a significant effect of feature type, *F*(2,34) = 47.74, *p <* 0.001, *pη*^2^ = 0.74. The episodic sentences had the highest vividness scores, then the perceptual sentences, and the functional sentences had the lowest vividness scores (*p’s <* 0.001). The effect of prior knowledge (*F*(1,17) = 7.48, *p =* 0.01, *pη*^2^ = 0.31) and the interaction between feature type and prior knowledge (*F*(2,34) = 6.54, *p =* 0.005, *pη*^2^ = 0.28) were significant. This was because the effect of prior knowledge was larger for the perceptual (*p =* 0.003) and functional sentences (*p =* 0.04) than the episodic sentences (*p =* 0.10).

**Living and nonliving categories**

In Experiment 1, to explore whether living/nonliving categories influence the effect of prior knowledge, we included the factor of animacy in the ANOVA analysis for the corrected recognition. The exemplars in familiar and unfamiliar categories were further divided as living (e.g., fruit, bird) and nonliving (e.g., tool, cloth) ones. The results showed that there were no significant effects of animacy (*F* (1,20) = 1.61, *p =* 0.22, *pη*^2^ = 0.08), neither interaction of prior knowledge, feature type and animacy (*F* (1,20) = 3.63, *p =* 0.08, *pη*^2^ = 0.15). There were no other significant interactions related to animacy (*F’*s < 2, *p’s >* 0.5). Importantly, the interaction of prior knowledge and feature type remained significant (*F* (1,20) = 7.72, *p =* 0.01, *pη*^2^ = 0.28). The results suggested that animacy may not influence the effect of prior knowledge on different feature types.

We also included the factor of animacy in the ANOVA analysis for the recall accuracy in Experiment 2. The results showed that there was a significant effect of animacy (*F* (1,23) = 18.89, *p =* 0.001, *pη*^2^ = 0.45), with sentences related to nonliving target words were recalled better than those related to the living target words. The interaction of prior knowledge, feature type and animacy was significant (*F* (1,23) = 5.40, *p =* 0.03, *pη*^2^ = 0.19). Further analysis showed that sentences with nonliving words showed significant effect of prior knowledge (*p*’s *<* 0.05). There were no other significant interactions related to animacy (*F’*s < 2, *p’s >* 0.5). The interaction of prior knowledge and feature type remained significant (*F* (1,23) = 6.57, *p =* 0.02, *pη*^2^ = 0.22), which suggested that animacy may not influence the effect of prior knowledge on different feature types.
